# Mental health and psychological impacts from the 2011 Great East Japan Earthquake Disaster: a systematic literature review

**DOI:** 10.1186/s40696-015-0008-x

**Published:** 2015-09-02

**Authors:** Nahoko Harada, Jun Shigemura, Masaaki Tanichi, Kyoko Kawaida, Satomi Takahashi, Fumiko Yasukata

**Affiliations:** 1 Division of Nursing, School of Medicine, National Defense Medical College, 3-2 Namiki, Tokorozawa, Saitama 359-8513 Japan; 2 William F. Connell School of Nursing, Boston College, Maloney Hall 140 Commonwealth Avenue, Chestnut Hill, MA 02467 USA; 3 Department of Psychiatry, National Defense Medical College, 3-2 Namiki, Tokorozawa, Saitama 359-8513 Japan

**Keywords:** Disaster, Mental health, Psychological service, Posttraumatic stress disorder, Great East Japan Earthquake, Earthquake, Tsunami, Fukushima Daiichi nuclear accident, Radiation fear

## Abstract

**Background:**

On March 11, 2011, Japan experienced an unprecedented combination of earthquake/tsunami/nuclear accidents (the Great East Japan Earthquake; GEJE). We sought to identify mental health and psychosocial consequences of this compound disaster.

**Method:**

A systematic literature review was conducted of quantitative research articles addressing mental health of survivors and the psychological impact of the GEJE. For articles between March 2011 and December 2014, PubMed, PsychINFO, and EMBASE databases were searched with guidance on literature review method.

**Results:**

Forty-nine studies met the inclusion criteria. A substantial proportion of the affected individuals experienced considerable psychological distress. Mental health outcomes included, but were not limited to, posttraumatic stress disorder, depression, and anxiety symptoms. Physical health changes, such as sleeping and eating disturbances, also occurred. In Fukushima, radioactive release induced massive fear and uncertainty in a large number of people, causing massive distress among the affected residents, especially among mothers of young children and nuclear plant workers. Stigma was additional challenge to the Fukushima residents. The review identified several groups with vulnerabilities, such as disaster workers, children, internally displaced people, patients with psychiatric disorders, and the bereaved.

**Conclusions:**

Following the GEJE, a considerable proportion of the population was mentally affected to a significant degree. The affected individuals showed a wide array of mental and physical consequences. In Fukushima, the impact of nuclear disaster was immense and complex, leading to fear of radiation, safety issues, and stigma issues.

## Background

**Fig. 1:**
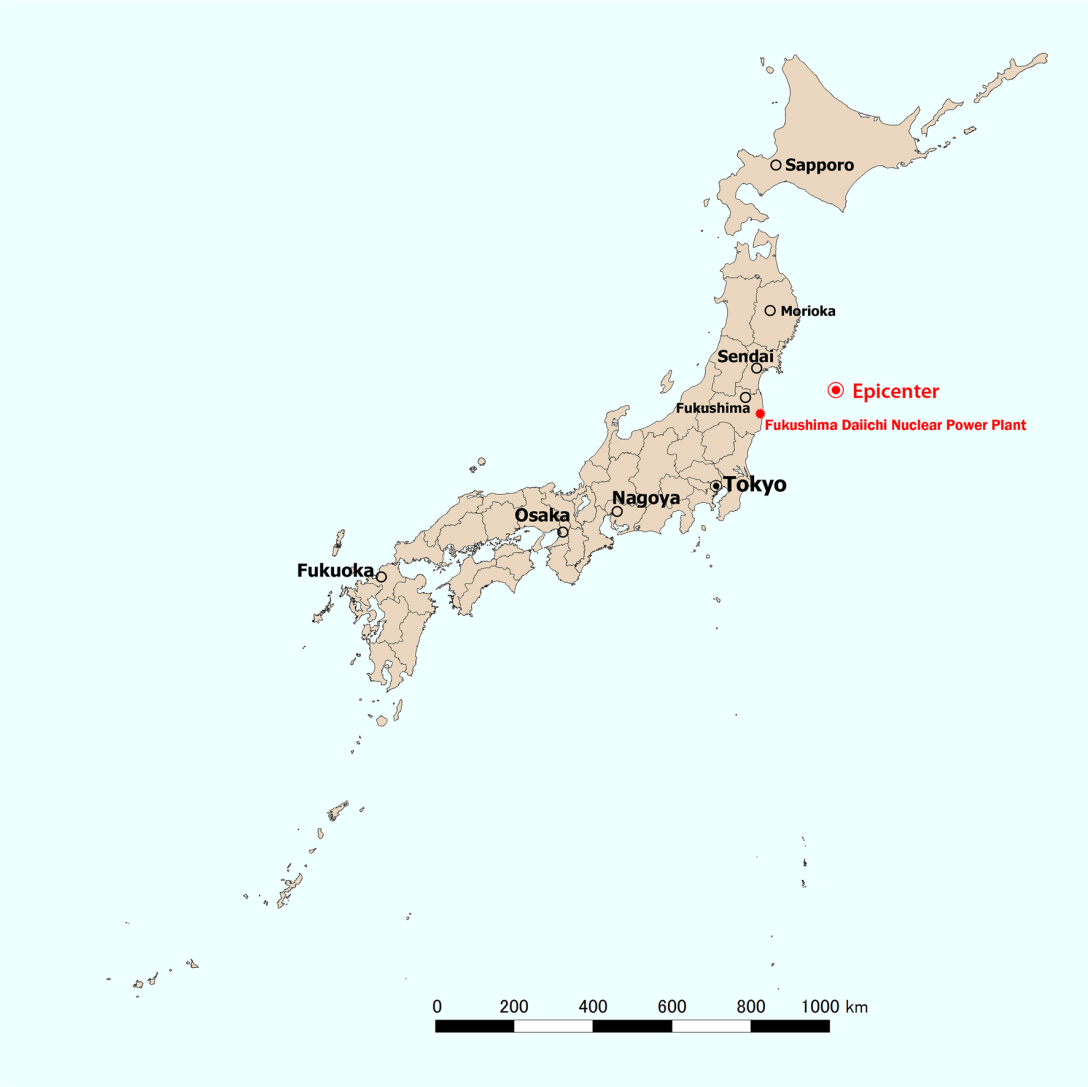
Locations of earthquake epicenter of the 2011 Great East Japan Earthquake and major cities

**Fig. 2:**
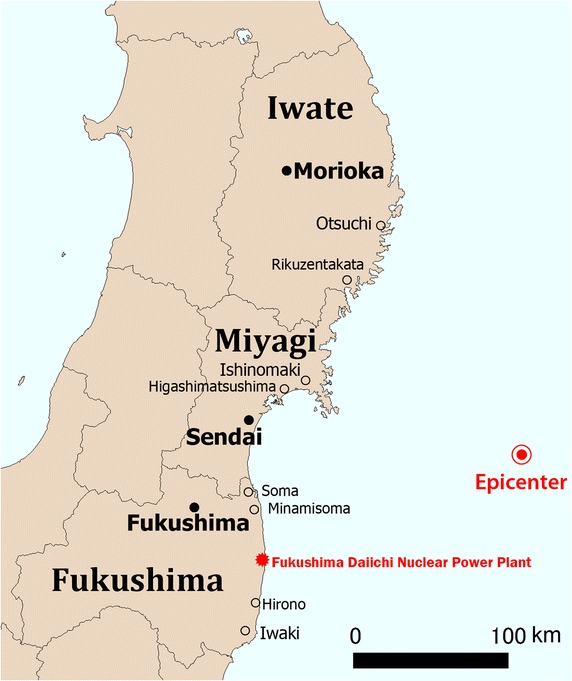
Three severely affected prefectures of the Tohoku region, Japan; Iwate, Miyagi, and Fukushima

On March 11, 2011, a 9.0-magnitude mega-earthquake hit the islands of Japan at 2:46 pm local time. This earthquake was the strongest recorded earthquake in the country’s modern history [1]. The earthquake’s epicenter was located approximately 80 km off the northeastern (Tohoku) region of the island of Honshu, the country’s main island. Repeated aftershocks and towering tsunami waves occurred after the mega-earthquake; the tsunami waves were as high as 40 m above sea level and reached 10 km inland (Fig. 1) [2]. A large majority of the damage occurred in three prefectures in the Tohoku area: Iwate, Miyagi, and Fukushima (Fig. 2). As of December 10, 2014, the numbers of dead, missing, or injured were 15,889, 2594, and 6152, respectively [3].

In Fukushima, the earthquakes and tsunamis triggered a nuclear accident at Tokyo Electric Power Company (TEPCO) Fukushima Daiichi Nuclear Power Plant. Between March 11 and 15, 2011, four of the six reactors experienced explosions, and three reactors escalated to nuclear meltdown and released radioactive materials, requiring a mandatory evacuation of the surrounding region. This crisis became the largest nuclear accident since the 1986 Chernobyl nuclear disaster, and the second accident, after Chernobyl, to measure Level 7 on the International Nuclear Event Scale.

On March 12, 2011, the Japanese government ordered mandatory evacuation of residents in the 20 km radius of the nuclear plant. As of December 2014, more than 121,000 residents are still on evacuation status [4]. Although there have been no fatalities owing to radiation exposure, safety concerns related to nuclear contamination created enormous fear, burden, and disruption to individuals, groups, communities, and local/national governments. These series of disasters were eventually named as the Great East Japan Earthquake (GEJE).

When evaluating post-disaster outcome studies, it must be noted every disaster is different in the terms of disaster type (e.g., natural, technological, manmade), location (e.g., developed vs. developing country), population (e.g., adults vs. children), intensity (life-threatening vs. non-), exposure frequency (e.g., single vs. repeated experiences), and many other medical/social/economic conditions. It is also very rare to randomize study samples or to have pre-disaster comparison [5].

Given these limitations, researches have shown a large majority of the people affected by disasters is resilient and will fully recover from their traumatic experiences [6]. A small portion of the affected individuals will result in a wide range of mental, behavioral, and physical health consequences [e.g., depression, posttraumatic stress disorder (PTSD) and other anxiety disorders, suicidal behaviors, alcohol misuse, and sleep disturbances]. Studies also have highlighted at-risk populations for adverse outcomes, such as female gender, preexisting psychiatric illnesses, presence of children in the home, secondary stressors, and low psychosocial resources [5–7]. Disaster workers, exposed to a variety of traumatic exposure through their work roles, are of significant concern when considering post-disaster mental health [5]. This trend was true for the Chernobyl first responders and clean-up workers as well; their depression and PTSD rates remained high two decades later [8].

A wide majority of these studies revealed the impact on mental health and psychosocial aspects of one particular event (e.g., man-made or natural). However, in the case of large-scale compound disasters, the epidemiological data are scarce. As of writing, GEJE mental health studies are evolving, but scientific review of this disaster’s mental health studies is warranted to increase better understanding of psychosocial outcomes of the people affected by the GEJE.

The aims of this article are to (1) consolidate quantitative and qualitative studies examining mental health and psychosocial impacts in people affected by the GEJE, (2) identify their mental health and psychosocial consequences, (3) ascertain vulnerable populations, and (4) elucidate factors that impact mental health and psychosocial outcomes in populations affected by the earthquake.

## Methods

A systematic literature review was conducted of quantitative research articles addressing mental health of survivors and the psychological impact of the 2011 GEJE between March 2011 and December 2014. PubMed, PsychINFO, and EMBASE databases were searched with guidance on literature review method [9]. The searched keywords included Great East Japan Earthquake Disaster, Japan, disaster, health, mental health, psychological, impact, stress, trauma, bereavement, and grief and these items were used either alone or in combination.

All identified articles were examined with the title and abstract whether the article specifically addresses mental health and psychosocial issues related to the GEJE by the investigators (NH and JS). If the abstract unclearly described the study aims, method, or results, NH and JS read the article to determine the relevance of the article. Studies written in a language other than English, situational reports, activity reports, conference reports/abstracts/summaries, letters to the editor (including replies), and bulletins from universities or private organizations were not included in this review. To ensure inclusiveness, the other authors of the current article were encouraged to search for articles manually and, if additional articles met the inclusion criteria, such articles were also included for review.

A total of 382 studies were identified, with 49 articles meeting the inclusion criteria. The identified articles were categorized by four main research topics: (1) mental health outcomes among affected populations (excluding Fukushima), (2) Fukushima resident studies, (3) disaster and support worker researches, and (4) grief studies. We decided to separate the first two topics because of the uniqueness of nuclear disaster and a potentially profound mental health impact among the affected people.

## Results

Table 1 summarizes the study results of mental health outcomes among the affected populations (excluding Fukushima). A total of 28 articles met this inclusion criterion [10–37]. A majority of the study populations were from Miyagi and Iwate [15 (53.6 %) and 3 (10.7 %), respectively]. Other studies included subjects from the Ibaraki, Tochigi, Tokyo, among others. A major portion of the studies were cross-sectional. Regarding outcome measures, nine (32.1 %) studies addressed PTSD, six (21.4 %) assessed general psychological distress, two (7.1 %) examined depressive symptoms. Other outcomes included anxiety, sleep disturbance, social functioning, social isolation, admission rates, suicide rates, and cerebral structure changes.

**Table 1:** Mental health outcomes among individuals affected by the GEJE (excluding Fukushima)

Citation (reference no.)	Sample type (location)	N	Data collection^a^	Findings (subject proportions, measurements)	Risk factors of outcome(s)
Yokoyama et al. [13]	Residents (Iwate)	10,025	6 and 11 months	42.6 %, K6 ≥ 5	Severe financial problems, displacement, lack of network
Niitsu et al. [12]	Residents (Iwate)	902	11 months	48 %, K6 ≥ 5	Female, middle-to-low educational status, unemployment
Koyama et al. [10]	Residents (Miyagi)	281	11 and 12 months	35.9 %, K6 ≥ 13	No social support, lower annual income, cohabitating with ≥6 people
Nagata et al. [11]	Residents of temporary housings (Iwate)	200	10–12 and 19–21 months	No significance in K6 ≥5 between two time points (37.5 %, 10–12 months vs. 43.5 %, 19–21 months). Sense of isolation higher at 19–21 months	
Sugimoto et al. [14]	National sample	8777	12 months	No significance in K6 ≥13 between certified vs. non-certified groups for house damage (8.4 vs. 9 %)	Lack of support from family, friends and neighbors
Fujihara et al. [15]	Diabetic patients (Ibaraki)	320	3 months pre- and post-disaster	Worsening of glycemic control was associated with total GHQ scores, interruption of drug regimen, somatic symptoms, and sleep disturbances/anxiety	
Inoue et al. [17]	Household of tsunami-affected houses (Miyagi)	4176	7–12 months	Social isolation	Men <65 years and living alone, low income
Funayama et al. [16]	Psychiatric outpatients (Tochigi)	294	2 months	4.1 % worsened and 1.2 % improved in GAF score	GAF score >50
Saito et al. [18]	Psychiatric in/outpatients (Tokyo)	155	1 week	3.5 % worsening of psychiatric symptoms (increase in epileptic seizure or GAF score)	
Aoki et al. [19]	Psychiatric patients on mandatory admission (Tokyo)	224	6 months pre- and post-disaster	Increased admission cases post-disaster (n = 127) compared to pre-disaster (n = 97)	Schizophrenia
Kato et al. 20]	Psychiatric inpatients with suicide attempt (Kanagawa)	592	6–1 months before and 1–6 months post-disaster	The number of admitted patients on ventilator was higher after the earthquake (Pre, n = 87 vs. post, n = 123)	Jobless, family psychiatric history, precipitating attempt, and alcohol intake
Orui et al. [23]	National government statistics report (Iwate, Miyagi and Fukushima)		24 months pre- and post-disaster	Suicide rates in men decreased during the post-disaster period; rates in women increased in the first 7 months	
Momma et al. [22]	Small and medium enterprise employees (Miyagi)	522	7 months pre- and 5 months post-disaster	14.3 % (men), IES-R ≥ 25 (5 months post-disaster) 24.4 % (women), IES-R ≥ 25 (5 months post-disaster)	Male: weak bilateral leg extension power, daily drinking habits, and depressive symptoms Female: hypertension and depressive symptoms
Takeda et al. [24, 25]	Female high school students (Miyagi)	1180	9 months	10 %, IES-R ≥25 Associated with premenstrual syndrome and premenstrual dysphoric disorder severity	
Iwadare et al. [21]	Junior high school student (Miyagi)	1919	8 and 20 months	Shorter sleep duration and later bedtime at 20 months	Bereavement experience
Usami et al. [36, 37]	Children, 4–15 years (Miyagi)	11,639	8 and 20 months	42.6 %, PTSSC-15 ≥ 23 at 8 months	Evacuation, house damage and/or separation from family, female, and not having breakfast
Kuwabara et al. [26]	Children, 6–15 years (Miyagi)	2259	6 months	Students from junior high schools with mortality rate ≥4 % had higher PTSSC-15 scores	
Numata et al. [31]	PTSD outpatients (Miyagi)	43	Unspecified	2.5 g of *saikokeishikankyoto* powder 3 times a day for 2 weeks improved IES-R score	
Tuerk et al. [35]	Residents (Ibaraki)	41	40 days post-event	27 %, self-reported PTSD symptoms	Subjective health and loss of sense of community
Niitsu et al. [29]	College students (USA)	30	12–14 months	Japanese students (n = 17) reported higher hyper-arousal than did non-Japanese students (n = 13)	Media exposure
Sekiguchi et al. [32]	Non-PTSD residents (Miyagi)	42	Pre-disaster, 3–4 months post-disaster	Regional volume changes in the brain observed after the disaster	Smaller GMV in the ACC before the earthquake, decreased GMV in the OFC through the earthquake
Sekiguchi et al. [33]	Non-PTSD residents (Miyagi)	30	Pre-disaster and 3–4 months post-disaster	Post-disaster anxiety level associated with cerebral structure changes	Lower FA in the right anterior cingulum, increased FA in the left anterior cingulum and uncinated fasciculus
Matsubara et al. [27]	Survivors remained at damaged residences (Miyagi)	5454	1–4 months	8.1 %, depressive reaction (PHQ-2)	House flooding below or above the ground floor, unavailability of gas supply, female, middle aged or elderly, regular intake of psychotropic medicine(s) since before the tsunami, no cohabitant
Nishigori et al. [30]	Postpartum women (Miyagi)	677	1 month pre- and 7 months post-disaster	20 %, Edinburgh Postnatal Depression Scale ≥9	Maternal age of under 25 years, child’s birth weight under 2.5 kg
Matsumoto et al. [28]	Residents (Miyagi)	4176	7–12 months	15 %, Sleep disturbance measured by an original scale	Lack of pleasure in life, lack of interaction with neighbors
Sugiura et al. [34]	Food delivery users (Tokyo and Osaka)	5053	2 months pre- and 1 months post-disaster	Insomnia (original scale), post- vs. pre-disaster odds ratio; Tokyo, 2.0, Osaka, 1.6	

*GEJE* Great East Japan Earthquake, *K6* Kessler Psychological Distress Scale, *GHQ* General Health Questionnaires, *GAF* global assessment of functioning, *IES-R* impact of event scale-revised, *PTSCC-15* posttraumatic stress symptoms for children 15 items, *GMV* grey matter volume, *ACC* anterior cingulate cortex, *OFC* orbitofrontal cortex, *FA* fractional anisotropy, *PHQ-2* Patient Health Questionnaire-2

^a^Cross-sectional studies unless otherwise noted

Table 2 compiles the 12 study results reporting psychosocial consequences of the individuals affected by the Fukushima nuclear disaster [38–49]. Adverse outcome measures were primarily general psychological distress, symptoms of PTSD, depression, as well as anxiety disorders, especially in context with radiation fear. Other outcomes represented the uniqueness of a nuclear disaster, such as concern of radiation and food safety [43, 49], maternal anxiety (including food safety, outdoor safety, radiation effects on embryos, economic issues, distrust towards information disclosure) [47, 49], lowered maternal confidence [47], and stigma owing to their radiation exposures [48].

**Table 2:** Mental health outcomes of Fukushima residents following the GEJE

Citation (reference no.)	Sample type (location)	n	Data collection^a^	Findings	Risk factors of outcome(s)
Matsumoto et al. [39]	Psychiatric outpatient (Fukushima)	1273	1 month	Bipolar I showed worst exacerbation among psychiatric diseases and manic change was prominent	
Wada et al. 2013 [45]	Psychiatric inpatient (Fukushima)	28	7 days	Two-thirds showed no change	
Yabe et al. [46]	Residents (Fukushima)	73,433 (2011), 39495 (2012)	10 and 22 months	14.6 vs. 11.9 %, K6 ≥13, 21.6 vs. 18.3 %, PCL ≥ 44 24.4 vs. 16.6 %, SDQ ≥ 16, 4–6 years 22.0 vs. 15.8 %, SDQ ≥ 16, 6–12 years (2011 vs. 2012)	
Tsubokura et al. [44]	Residents (Fukushima)	155	1 year pre- and post-disaster	12 %, PHQ-9 ≥ 10	
Kukihara et al. [38]	Evacuees (Fukushima)	241	9 months	33.2 %, IES-R ≥ 25 14.5 %, Zung Depression Scale ≥60 Resilience was predicted by employment status, eating/exercise and drinking habits	
Sawa et al. [41, 42]	Internally displaced people from Fukushima (Chiba)	73	5 and 10 months	Compared to a reference group (Chiba residents), the study sample was more likely to have GHQ-12 ≥ 3, adverse social dysfunction at both time points and psychological distress at 10 months	
Sugimoto et al. [43]	National sample	5809	1 year	Women were more concerned than men about radiation. Participants from Kanto region (vs. non-Tohoku/Kanto regions) were more concerned about radiation and food safety	
Rubin et al. [40]	British nationals living in Japan	284	9 mo.	16 %, GHQ-12 ≥ 4 29.7 %, State-trait anxiety inventory ≥ 18 30.4 %, State-trait anger inventory ≥ 11	
Goto et al. [47]	Parenting counseling records (Fukushima)	150	1 year pre-, 1 and 13 months post-disaster (qualitative analysis)	Lowered maternal confidence and potential role of public health nurses in risk communication process post-disaster were reported	
Yoshii et al. [49]	Post-partum women (Miyagi)	259	16 months (qualitative analysis)	Seven themes of maternal anxiety for radioactivity from the Fukushima emerged: food safety, outdoor safety, radiation effects on embryos during pregnancy, effects on children, radiation exposures, economic issues and distrust towards disclosing information	
Ben-Ezra et al. [48]	Residents (Hiroshima/Nagasaki, Tokyo and Fukushima)	750	3 years post-disaster	10.6 %, Fukushima, endorsed PTSD symptoms 2.4 %, Hiroshima/Nagasaki and Tokyo, endorsed PTSD symptoms Relations between location, radiation stigma, and number of PTSD symptoms	

*GEJE* Great East Japan Earthquake, *K6* Kessler Psychological Distress Scale, *PCL* PTSD checklist, *SDQ* Strengths and Difficulties Questionnaire, *PHQ-9* Patient Health Questionnaire-9, *IES-R* impact of events-revised, *GHQ-12* General Health Questionnaires 12

^a^Cross-sectional studies unless otherwise noted

Table 3 represents eight study outcomes of GEJE disaster and support workers [50–57]. Their outcomes measures were general psychological distress as well as symptoms of PTSD or depression. Of note, a study of Fukushima nuclear plant workers showed discrimination/slurs experience as a key factor for their mental health consequences [56]. This trend was associated with the public criticism to the electric company’s post-disaster management. One study focused on potential of fish oil in attenuating PTSD symptoms among DMAT (Disaster Medical Assistant Team) medical workers [53].

**Table 3:** Studies of the GEJE disaster and support workers

Citation (reference no.)	Sample type (location)	n	Data collection^a^	Findings	Risk factors of outcome(s)
Shigemura et al. [55, 56]	Nuclear plant workers (Fukushima)	1495	2–3 months	42.7 %, K6 ≥ 13 25.3 %, IES-R ≥ 25	Preexisting illness(es), discrimination/slurs, near-death experience, tsunami evacuation, major property loss, home evacuation
Dobashi et al. [50]	Defense personnel (Miyagi)	606	1 month post deployment	6.2 (±8.1), IES-R 12.8 (±4.4), K10	No identified factors
Matsuoka et al. [52]	Disaster Medical Assistant Team	426	1 month	4.0 %, K6 ≥ 13 21.4 %, CES-D ≥ 17	Concern over radiation exposure
Nishi et al. [54]	Disaster Medical Assistant Team	173	4 months	6.8 (±8.4), IES-R	PDI score and watching earthquake TV news reports ≥4 h/day
Nishi et al. [53]	Disaster Medical Assistant Team	172	Baseline and 12-week post-intervention	Fish oil attenuated posttraumatic stress symptoms among female	
Fukasawa et al. [51]	Government workers (Miyagi)	4331	2 months	3.0 %, K6 ≥13 (group with less property damages) 5.9 %, K6 ≥ 13 (group with severe property damages)	Less damaged: working overtime (>100 h/mo. overtime), poor workplace communication Severe damaged: handling residents’ complaints, poor workplace communication
Suzuki et al. [57]	Government workers (Miyagi)	3743	7 months	9.6 %, K6 ≥ 10 4.4 %, K6 ≥ 13	Not taking a non-work day each week

*GEJE* Great East Japan Earthquake, *K6 (K10)* Kessler Psychological Distress Scale, *IES-R* impact of events-revised, *CES-D* Center for Epidemiologic Studies Depression Scale, *PDI* peritraumatic distress inventory

^a^Cross-sectional studies unless otherwise noted

Table 4 shows a result of a sole study examining the grief responses affected by the GEJE [58]. This study showed the distinctiveness of complicated grief from symptoms of PTSD or depression. Other articles, not listed in the table, were leaned on narratives and support activity reports. For example, our co-author (ST) launched a support group immediately after the disaster for people in grief and bereavement [59]. This support group aims to (1) provide information about grief, the concept of which is not as popularly known in Japan (much like PTSD), and (2) provide training sessions and workshops in collaboration with the local grief support organizations.

**Table 4:** Grief study following the GEJE

Citation (reference no.)	Sample type (location)	N	Data collection^a^	Findings	Risk factors of outcome(s)
Tsutsui et al. [58]	Hospital workers (tsunami-affected area)	82	8 months	9.8 %, ICG ≥ 25 29.3 %, IES-R ≥ 25 37.8 %, CES-D ≥ 16 Prolonged grief disorder in qualitatively distinct from PTSD and major depressive disorder	

*GEJE* Great East Japan Earthquake, *ICG* Inventory of Complicated Grief, *IES-R* impact of events-revised, *CES-D* Center for Epidemiological Studies Depression Scale

^a^Cross-sectional studies unless otherwise noted

## Discussions

Our review compiled a wide array of mental health consequence following the GEJE, an unprecedented compound disaster with a combination of earthquakes, tsunamis, and a series of nuclear accidents. A considerable proportion of the study population was mentally affected to a substantial degree, and mental health responses ranged from approximately one-tenth to nearly half of the respondents [12, 27]. Mental health outcomes included, but were not limited to, PTSD, depression, and anxiety. Physical health changes, such as sleep and eating disturbances, were also reported.

Although every disaster is different, disasters are large-scale, stressful, and distressing events that affect a significant number of people. Those who experience higher exposure to traumatic events are likely to show higher mental health responses (i.e., dose–response relationship) [60]. For the most people, these acute responses are normal and gradually decrease over time, but a small proportion of the affected individuals will suffer long-term mental health issues. In a review of 160 disaster mental health studies, proportions of subjects with severe impairment were 21.6 % for natural disasters and 18.5 % for technological disaster samples [6]. The articles in this review had relatively higher mental health rates than in previous studies. This trend might be related to the high impact of this disaster as well as the GEJE study timing, because most of the studies were conducted among the direct victims within 2 years after the disaster. Long-term, longitudinal studies are evolving, and they will potentially be useful to understand the trajectories of mental health consequences among these people.

In the region affected by the Fukushima nuclear disaster, invisible and imperceptible nature of radioactive materials has been challenged among the affected people. The residents’ responses were diverse and complex; along with high proportions of mental health distress among the Fukushima residents [46], concerns for radiation effect were a prominent concern especially among pregnant women and mothers of young children [43, 49, 61]. Safety issues in food and outdoor activities, along with economic issues and distrust in information disclosure were also reported [43, 49]. Public psychosocial responses such as discrimination and stigmatization were also reported [48, 56].

These findings are in accordance with a series of Chernobyl studies where a complex relationship between radiation exposure and physical/mental health effect has been an ongoing debate. Physical outcome studies tend to be controversial, although firm evidence can be found only on the deaths of first responders due to acute radiation exposures and high prevalence of thyroid cancer among the exposed children [8]. Still, psychosocial and economic disruptions to the affected people were significant, and the International Atomic Energy Agency regarded mental health as the major public health sequelae of the Chernobyl accident [62]. Mothers of young children and plant clean-up workers were among the two groups of particular concern [63–65]. Psychosocial issues included not only mental health disorders but also stigmatization and discrimination of the affected people [8], suggesting the importance of integrity and accuracy of information as well as risk communication strategies.

Two Fukushima studies reported distress among internally displaced people [38, 41]. Mandatory evacuation measures have been in place for the area surrounding the nuclear plant, and the evacuees potentially have uncertain and ambiguous perspectives on whether or not they will be able to return home [66]. This trend was also compatible with Chernobyl studies reporting challenges in evacuation and resettlement [8]. Future studies will be essential to clarify the effect of evacuation following nuclear disasters.

Fukushima and Chernobyl studies suggest that substantial public health efforts are crucial to establish a system capable of such exposures. Integrity and accuracy of information will be a critical issue for the public to assess their health status. These studies also have implications for other “tangible” disasters, such as emergencies related to bio-chemical weapons and infectious diseases [67]. Long-term studies will be important to increase the psychosocial impact among Fukushima residents, with special focus on children, mothers, and nuclear plant workers.

A number of studies assessed a considerable degree of mental distress among disaster workers. This is likely to be owing to work-related exposures of these workers. In the case of GEJE, many of the workers were also local disaster victims, and had struggles as survivors along with their work-related exposures. This effect was prominent in several studies [51, 56, 57]. Two worker studies identified experiences of being discriminated against and handling residents’ complaints as risk factors for their adverse mental health [56, 57]. In the former study, the Fukushima nuclear plant workers became targets of public criticism because their company was blamed for their post-disaster mismanagement. In the latter, Miyagi Prefecture workers received direct complaints from their residents in a chaotic situation. These results might give hypotheses that mental health of disaster workers is susceptible to their stakeholder’s criticisms.

Past literatures identified mortuary work as predictors of PTSD or physical symptoms among disaster workers [68, 69], but in our review, there has yet be an evidence that mortuary work was associated with adverse mental health [50, 51]. Further studies will be needed to elucidate the relationship between dead body exposure and mental health outcomes among this population.

Previous studies highlighted vulnerable populations for post-disaster mental health, such as children, evacuees, the bereaved, and individuals with preexisting physical/mental health conditions [5, 6]. Our compilation overall shows a similar trend, although studies are relatively few, especially in the context of grief.

We recognize several limitations of this paper. The GEJE, especially the Fukushima nuclear accident, is an ongoing disaster, and new studies are emerging. Given the timing and methodology of our literature search, we were not able to include narrative studies, non-English papers, or papers describing long-term disaster impact. Although we made every possible effort to include all related studies, some studies may have been inadvertently omitted.

Given these limitations, this literature review encompasses research on the mental health trajectories of people affected by the GEJE, a complex earthquake/tsunami/nuclear disaster. Along with our review, future studies will be essential for having a better understanding of this disaster, and especially for ascertaining the long-term outcomes and their correlates.
